# Corneal Nerve Injury and Regeneration: Mechanisms, Clinical Consequences, and Emerging Therapeutic Strategies

**DOI:** 10.1007/s40265-026-02396-w

**Published:** 2026-10-08

**Authors:** Athar Shadmani, Anat Galor, Houri Esmaeilkhanian, Maja Kostic

**Affiliations:** 1 Bascom Palmer Eye Institute, University of Miami Miller School of Medicine, 900 NW 17th Street, Miami, FL 33136 USA; 2 Surgical Services, Miami Veterans Affairs Medical Center, Miami, FL USA; 3 Byers Eye Institute, Stanford School of Medicine, Palo Alto, CA USA; 4 School of Medicine, Saint Louis University, Saint Louis, MO USA

## Abstract

The cornea is among the most densely innervated tissues in the body and depends on intact sensory and autonomic innervation to maintain epithelial integrity, tear film homeostasis, and ocular surface defense. Corneal nerve injury may occur after surgery, infection, inflammation, trauma, or systemic disease and can lead to reduced corneal sensitivity, impaired epithelial healing, dry eye, neuropathic pain, and neurotrophic keratopathy. Effective management requires both treatment of the underlying cause and restoration of a microenvironment permissive for reinnervation. This review examines the mechanisms underlying corneal nerve injury and repair, with emphasis on therapeutic strategies relevant to clinical translation. These include anti-inflammatory and immunomodulatory agents, growth factors and cytokines, blood-derived products, extracellular matrix-based therapies, stem cell- and secretome-based approaches, surgical neurotization, and physical stimulation modalities. We also highlight emerging challenges in the field, including the gap between structural nerve regeneration and functional recovery, limitations in clinical evidence, and barriers to therapeutic accessibility and implementation.

## Key Points

**Table:** 

Corneal nerve injury can lead to a range of adverse outcomes, including epithelial breakdown, dry eye, neuropathic pain, and neurotrophic keratopathy (NK), with manifestations varying by the cause of injury and the individual response to injury.
Therapeutic strategies for corneal nerve regeneration include anti-inflammatory agents, neurotrophic factors, biologics, extracellular matrix-based therapies, cell-free regenerative approaches, and surgical neurotization.
Future progress will depend on therapies that improve not only nerve structure, but also durable functional recovery, clinical feasibility, and access.

## Introduction

The ocular surface is innervated by both sensory and autonomic components of the nervous system, which together maintain corneal integrity, transparency, and surface homeostasis [1]. The cornea is predominantly supplied by sensory fibers, with a density estimated to be 300–600 times greater than that of skin, making it one of the most sensitive tissues in the human body [2]. Corneal sensory nerves rapidly detect environmental stimuli and initiate protective reflexes, while also contributing to epithelial repair and inflammatory regulation [1]. Sensory input from the cornea and conjunctiva is conveyed through the ophthalmic division of the trigeminal nerve (CN V1).

Autonomic innervation also has an important role in ocular surface maintenance. Sympathetic fibers reach the eye via the posterior ciliary nerves and limbal vascular plexus [2], whereas parasympathetic fibers travel with the facial nerve and ultimately provide secretomotor input to the lacrimal gland, conjunctival goblet cells, and accessory lacrimal glands [1, 3]. Autonomic nerves system also contributes to epithelial renewal and local immune regulation through macrophage-mediated pathways [4]. Together, these pathways regulate tear secretion and support tear film stability, lubrication, and nutrient delivery [1, 5]. Meibomian glands and conjunctival goblet cells also respond to autonomic neurotransmitters that regulate lipid and mucin secretion [6].

After entering the cornea, nerves lose their perineurium and myelin sheath near the limbus and form stromal, subepithelial, and subbasal plexuses that are essential for epithelial integrity and function [3]. Beyond sensory transmission, corneal nerves exert trophic and immunomodulatory effects through the release of neuropeptides and neuromediators. Substance P (SP) promotes corneal epithelial stratification and keratocyte migration and may contribute to epithelial repair [7, 8]. Both SP- and calcitonin gene-related peptide (CGRP)-positive nerve fibers are distributed throughout the corneal innervation [9]. Calcitonin gene-related peptide released from corneal sensory nerves also contribute to lacrimal-gland tear secretion [10]. Calcitonin gene-related peptide additionally modulates innate immune responses by suppressing pro-inflammatory cytokine production in macrophages and dendritic cells [11].

Corneal epithelial cells also support nerve survival and regeneration by producing neurotrophic factors, including nerve growth factor (NGF), neurotrophin-3, neurotrophin-4/5, epidermal growth factor, brain-derived neurotrophic factor (BDNF), and glial cell line-derived neurotrophic factor [12]. Ciliary neurotrophic factor (CNTF) is also present in the cornea, although it is not exclusively epithelial in origin [13]. These observations support reciprocal trophic interactions between corneal epithelium and nerves [12–14].

All these interconnected mechanisms that maintain corneal clarity can be disrupted by various nerve injuries, including metabolic and inflammatory diseases, infectious keratitis, ocular surgery, chemical trauma, prolonged contact-lens wear, topical drug toxicity, and trigeminal or neurosurgical injury [1].

With the increasing availability of biologics, regenerative therapies, and surgical interventions, corneal nerve regeneration has emerged as a clinically actionable therapeutic target rather than a purely descriptive concept. This review therefore focuses on current and emerging therapeutic strategies that support restoration of corneal nerve health, their mechanisms, current evidence base, and translational relevance.

## Literature Search Strategy

This narrative review was informed by a literature search of PubMed/MEDLINE, Scopus, and Google Scholar for articles related to corneal nerve injury and regeneration from database inception through August, 2026. Search terms included combinations of “corneal nerve regeneration,” “neurotrophic keratopathy (NK),” “corneal innervation,” “cenegermin,” “amniotic membrane,” “regenerative therapy,” “electrical stimulation,” “extracellular matrix,” and “corneal neurotization.” Relevant English-language original studies, reviews, and clinical reports were selected, with emphasis on recent translational and clinical literature, while additional references were identified from the bibliographies of key articles.

## Pathologies with Corneal Nerve Damage

A broad range of systemic and local insults can disrupt corneal homeostasis by damaging sensory nerves and autonomic pathways from their central origins to intra-corneal terminals. Under pathological conditions, sympathetic fibers may aberrantly sprout into the cornea, as observed after herpes simplex stromal keratitis and in experimental models of sensory denervation [15].

Following corneal injury, sympathetic activation promotes neutrophil infiltration and pro-inflammatory cytokine release, thereby impairing re-epithelialization, whereas parasympathetic activation suppresses inflammation and facilitates epithelial repair [4]. Crosstalk between the autonomic nervous system and mast cells also appears important for regulating inflammation and coordinating wound healing [16].

In systemic disorders such as diabetes, autonomic dysfunction contributes to tear film instability, sensory nerve impairment, and neuroinflammation, with downstream consequences that include delayed corneal repair, neuropathic pain, NK, band keratopathy, and progressive vision loss [17, 18]. The principal causes of corneal nerve damage are summarized below.

### Inflammatory and Metabolic Conditions

A limited inflammatory response is necessary for repair and may promote corneal nerve regeneration [14]. However, persistent or excessive inflammation, particularly involving interleukin (IL)-1 and tumor necrosis factor (TNF)-α, impairs nerve recovery and sustains neuroinflammatory injury [17, 18]. As such, it is not surprising that systemic diseases involving chronic inflammation, such as Sjögren’s disease, are associated with nerve abnormalities [19]. In vivo confocal microscopy (IVCM) studies have demonstrated reduced or abnormal central subbasal nerve parameters in Sjögren’s disease [19]. Multiple sclerosis is another inflammatory disease that has been shown to impact corneal nerves [20]. Metabolic disease, particularly diabetes, can impact nerves through several mechanisms, including alterations in neuronal metabolism and changes in the surrounding neural microenvironment. Together, these mechanisms can impair axonal transport, reduce Schwann cell support, damage the small vessels that nourish nerves, and create a pro-inflammatory environment that limits repair [21, 22]. Diabetes-related injury to trigeminal sensory pathways can lead to reduced corneal innervation and impaired epithelial healing [23]. Diabetes is also associated with increased corneal Langerhans cells and dendritic cells, which correlate inversely with nerve fiber density, linking immune activation to neural loss [22].

### Infection

Herpetic infections, particularly herpes simplex virus type 1 and varicella-zoster virus, are among the most important infectious causes of corneal sensory nerve injury and hypoesthesia [24]. Herpes viruses are neurotropic and can injure sensory nerves through direct neuronal infection, retrograde transport to sensory ganglia, latency/reactivation, and immune-mediated inflammation, ultimately reducing corneal innervation and impairing epithelial trophic support. In a population-based cohort study, the annual incidence of new herpes simplex virus (HSV) keratitis was 9.2 per 100,000 persons, while the cumulative probability of recurrent ocular HSV disease after an initial episode was 27% at 1 year, 50% at 5 years, and 63% at 20 years [25]. Varicella-zoster virus similarly remains latent in the trigeminal ganglion and may reactivate with ocular involvement, often as herpes zoster ophthalmicus [26]. Beyond herpes, parasitic and fungal keratitis can also cause severe corneal nerve injury through both direct invasion and host inflammatory responses. Acanthamoeba is particularly notable for direct neural infiltration, radial neuritis, and severe pain [27]. Systemic infections, such as leprosy, are also recognized causes of NK [28].

### Trauma

Traumatic corneal nerve injury commonly follows refractive surgery and other anterior segment procedures, which predictably damage stromal nerves and the subbasal plexus [29]. Corneal nerve abnormalities may also occur after chemical injury, with prolonged contact-lens wear, particularly orthokeratology, or following chronic exposure to preservative-containing topical medications [30, 31].

### Medications

Many systemic medications, including neuroleptics, antipsychotics, and antihistamines, may impair trigeminal nerve function and contribute to corneal abnormalities [18]. As noted above, topical medications, especially those containing benzalkonium chloride (BAK), have also been shown to adversely affect corneal nerve health, likely through direct epithelial and neural toxicity, tear film disruption, oxidative stress, and local inflammation [31].

### Neurologic or Neurosurgical Conditions

Neurologic or neurosurgical conditions can damage the corneal nerves when the trigeminal nerve or its ophthalmic branch is injured, because these fibers provide the main sensory innervation to the cornea. Trigeminal neuralgia surgery, acoustic neuroma surgery, brainstem or trigeminal pathway lesions, stroke, tumors, or trauma can interrupt corneal sensation and reduce trophic support to the epithelium. As a result, patients may develop decreased corneal sensitivity, impaired epithelial healing, and persistent epithelial defects [18, 32].

### Ocular Surface Diseases

Ocular surface diseases such as limbal stem cell deficiency (LSCD) and cicatrizing conjunctival disease can damage corneal nerves through chronic epithelial breakdown, inflammation, fibrosis, and disruption of the normal limbal–corneal microenvironment. Persistent surface inflammation and scarring may reduce subbasal nerve density and branching, leading to decreased corneal sensitivity and impaired epithelial healing. This nerve damage can further worsen ocular surface instability and contribute to a cycle of delayed healing and neurotrophic changes [24, 33].

## Corneal Nerve Regeneration Enhancement

**Fig. 1:**
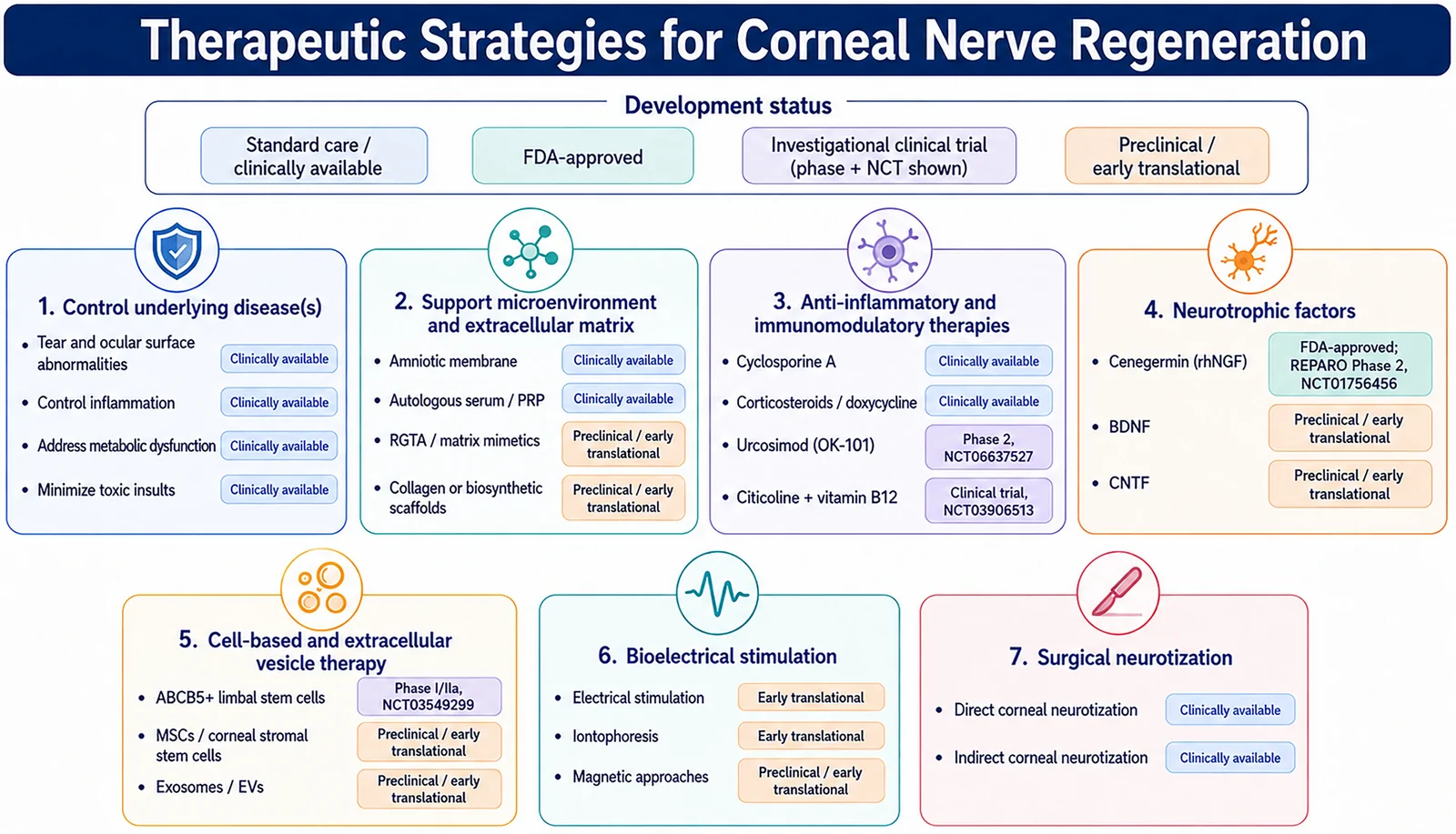
Therapeutic strategies to promote corneal nerve regeneration. Therapeutic approaches optimize the ocular surface environment and enhance corneal nerve repair and include control of underlying disease, extracellular matrix support, neurotrophic factors, cell-based/extracellular vesicle therapy, bioelectrical stimulation, and surgical neurotization. *BDNF* brain-derived neurotrophic factor, *CNTF* ciliary neurotrophic factor, *EVs* extracellular vesicles, *MSCs* mesenchymal stem/stromal cells, *NCT* ClinicalTrials.gov identifier, *PRP* platelet-rich plasma, *RGTA* regenerating agent

Therapeutic strategies for corneal nerve regeneration can be broadly divided into approaches that improve the ocular surface microenvironment and those that promote neural repair. Table 1 summarizes therapies in clinical practice or clinical translation for corneal nerve regeneration according to therapeutic class, proposed mechanism, translational status, and major limitations. Figure 1 categorizes the mechanism of different modalities for enhancement of corneal nerve regeneration.

**Table 1:** Current and investigational approaches to corneal nerve regeneration

Therapeutic class	Therapy/example	Development status	Main mechanism	Key limitation
*A. FDA-approved or clinically available therapies*				
Anti-inflammatory/immunomodulatory agents	Cyclosporine A; corticosteroids; doxycycline	Clinically available; use for corneal nerve regeneration is indirect and generally off-label	Suppress maladaptive inflammation and improve ocular surface conditions permissive for nerve recovery	Direct neuro-regenerative effect and durability remain uncertain
Growth factor	Cenegermin (rhNGF)	FDA-approved for neurotrophic keratitis; pivotal Phase 1/2 REPARO trial, NCT01756456	Promotes epithelial healing, neuronal survival, axonal growth, and corneal sensitivity	High cost, limited access, treatment burden, and incomplete functional recovery
Blood-derived products	Autologous serum tears; PRP; PRGF	Clinically used in selected settings; not specifically approved for corneal nerve regeneration	Provide growth factors, cytokines, and trophic proteins	Variable preparation, limited standardization, and reimbursement issues
Extracellular matrix-based therapy	Amniotic membrane	Clinically available for ocular surface repair; nerve-regenerative benefit supported by limited studies	Provides anti-inflammatory, matrix-supportive, and epithelial-trophic effects	Small studies, product variability, and uncertain direct neural effect
Surgical neurotization	Direct nerve transfer; inter-positional sural or great auricular nerve grafts	Specialized clinical use	Restores sensory axonal input to the anesthetic cornea	Technically complex; limited access; donor-site morbidity; variable recovery
*B. Investigational and emerging therapies*				
Anti-inflammatory/immunomodulatory agent	Urcosimod (OK-101)	Investigational; Phase 2a trial terminated early after enrolling 18 participants; NCT06637527	ChemR23 agonism with anti-inflammatory and analgesic effects; potential improvement in corneal nerve metrics	Available efficacy data are company-reported; peer-reviewed evidence and durability remain limited
Neuroprotective combination	Topical citicoline plus vitamin B12	Investigational; randomized pilot clinical trial, NCT03906513 (Phase not specified)	May support neuronal membrane metabolism, cellular recovery, and axonal repair	Small sample and single study; generalizability remains uncertain
Growth factors/cytokines	BDNF; CNTF	Preclinical / early translational; no applicable NCT identified	Support neuronal survival, differentiation, and axonal regrowth	No established human therapeutic efficacy
Blood-derived products	Umbilical cord blood serum; umbilical cord blood platelet lysate	Investigational/early translational; no applicable NCT identified	Provide allogeneic epithelial-supportive and neurotrophic mediators	Product variability, manufacturing, immunologic, and regulatory challenges
Extracellular matrix-based therapies	Heparan sulfate analogs (RGTA); collagen or biosynthetic scaffolds	Early translational/preclinical; no applicable NCT identified	Stabilize extracellular matrix and growth factors and provide a substrate for epithelial repair and neural ingrowth	Nerve-specific benefit remains incompletely defined
Stem cell-based therapy	ABCB5-positive limbal stem cells	Investigational; Phase 1/2a, NCT03549299 (LSCD trial; indirect relevance to nerve regeneration)	Restores epithelial integrity and local trophic support	Direct corneal nerve regeneration has not been established
Stem cell-based therapies	MSCs; corneal stromal stem cells	Preclinical/early translational; no applicable NCT identified	Mainly paracrine immunomodulatory and trophic effects	No clear evidence of direct differentiation into functional corneal nerves
Cell-free regenerative therapies	MSC-derived exosomes; extracellular vesicles	Preclinical; no applicable NCT identified	Deliver bioactive proteins, lipids, and RNA to modulate inflammation and repair	Dosing, cargo variability, manufacturing, and long-term safety remain unresolved
Physical stimulation/device-based therapy	Electrical stimulation; trigeminal or nasociliary stimulation	Preclinical/early translational; no applicable NCT identified	Upregulates neurotrophic factors and may support tear secretion and epithelial healing	Limited clinical evidence and no standardized treatment protocols
Physical stimulation/device-based therapy	Iontophoresis	Early translational; no corneal nerve-regeneration trial identified	Enhances ocular drug penetration and may provide electrical trophic effects	Observed benefit may reflect improved drug delivery rather than direct neuro-regeneration

Clinical trial phase and NCT identifier are reported where applicable

*ABCB5* ATP-binding cassette subfamily B member 5, *BDNF* brain-derived neurotrophic factor, *CNTF* ciliary neurotrophic factor, *FDA* US Food and Drug Administration, *LSCD* limbal stem cell deficiency, *MSCs* mesenchymal stem/stromal cells, *NCT* ClinicalTrials.gov identifier, *PRGF* plasma rich in growth factors, *PRP* platelet-rich plasma, *RGTA* ReGeneraTing Agent, *rhNGF* recombinant human nerve growth factor, *RNA* ribonucleic acid

### Therapies Targeting the Ocular Surface Microenvironment

#### Mechanism of Action

The corneal extracellular microenvironment provides structural and biochemical signals that influence epithelial repair, axonal guidance, and cellular responses during nerve regeneration. Extracellular matrix components, including heparan sulfate, laminin, fibronectin, hyaluronic acid, and collagen, contribute to growth factor retention, epithelial adhesion, and neural remodeling. Therefore, therapeutic strategies that preserve or reconstruct this microenvironment may indirectly enhance corneal nerve repair by reducing epithelial stress, stabilizing trophic signaling, and creating a substrate permissive for reinnervation.

#### Available Therapies

Amniotic membrane transplantation is a clinically established matrix-based therapy that provides trophic, anti-inflammatory, anti-fibrotic, and epithelial-supportive effects [34]. From a drug-focused and translational perspective, its value lies less in acting as a single pharmacologic agent and more in delivering a biologically active matrix that can modulate inflammation, support epithelial repair, and potentially promote neural recovery. In a prospective randomized study of 20 patients with moderate-to-severe dry eye disease, a self-retained cryopreserved amniotic membrane was applied for approximately 3–5 days, and patients were followed for 3 months [35]. Corneal sensitivity increased from 3.25 ± 0.60 cm at baseline to 5.20 ± 0.50 cm at 1 month and 5.60 ± 0.40 cm at 3 months. The Dry Eye Workshop (DEWS) severity score decreased from 2.9 ± 0.3 at baseline to 1.1 ± 0.3 and 1.0 at 1 and 3 months, respectively, accompanied by increased central corneal nerve density on IVCM. These findings suggest a potential regenerative effect, although the small sample and short follow-up limit generalizability. This study supports a potential regenerative role beyond epithelial resurfacing alone in at least one condition associated with corneal nerve abnormalities (i.e., dry eye).

Blood-derived therapies are another approach used to support a healthier ocular surface microenvironment. Blood-derived therapies provide a complex mixture of growth factors, cytokines, and bioactive proteins that may support both epithelial recovery and nerve regeneration. Platelet-rich plasma (PRP) and autologous serum tears (AST) are already used clinically for severe ocular surface disease, particularly refractory epithelial defects, and dry eye disease [36, 37]. In a prospective, nonrandomized study of 368 patients with moderate-to-severe dry eye disease, 6 weeks of autologous PRP monotherapy improved symptoms in 322 patients (87.5%) and was associated with reductions in corneal fluorescein staining and improvements in several ocular-surface measures [36]. However, the study did not assess corneal nerve density or sensitivity; therefore, it supports ocular-surface improvement rather than direct neuroregeneration.

Plasma rich in growth factors (PRGF) has also been used in NK and has been associated with improvements in epithelial integrity, corneal sensitivity, and visual acuity [38]. In a retrospective study of 38 eyes with stage 2–3 neurotrophic keratitis, PRGF eye drops achieved complete corneal ulcer/defect closure in 97.4% of cases, with a mean healing time of 11.4 weeks [38]. Treatment was associated with significant improvements in ocular-surface symptoms and best corrected visual acuity (BCVA), with only one reported adverse event (itchy eye feeling) (2.6%), supporting PRGF as a potentially safe therapeutic option. In a small case series of four patients with severe NK secondary to herpes zoster ophthalmicus, treatment with 20% autologous serum tears eight times daily was associated with restoration of the subbasal nerve plexus on in vivo confocal microscopy within 3–7 months, accompanied by partial recovery of corneal sensation [39].

#### Developing Therapies

Developing blood-derived therapies aim to improve the standardization and scalability of serum- and platelet-derived formulations. Allogeneic approaches, including umbilical cord blood serum and umbilical cord blood platelet lysate, are being investigated as alternatives when AST or PRP is impractical or biologically suboptimal. These products may provide higher concentrations of epithelial supportive and neurotrophic mediators, but larger controlled trials with nerve-specific outcomes are still needed [40].

Another area of research involves regenerative agents that act as heparan sulfate analogs to stabilize extracellular matrix architecture and prolong local growth factor availability [41]. One such compound is ReGeneraTing Agent (RGTA), which has been studied in persistent epithelial defects, neurotrophic ulcers, corneal ulcers, and severe infectious keratitis-related nonhealing epithelial defects. In one case series of 27 eyes with persistent epithelial defects after severe infectious keratitis, RGTA treatment achieved complete epithelial healing in all patients within a mean of 2.36 ± 1.64 weeks, without reported local or systemic side effects [42].

Synthetic heparan sulfate mimetic have also been studied in animal models. In a mouse corneal laser-ablation model, synthetic heparan sulfate mimetic polymer (HSmP) improved epithelial architecture, reduced myofibroblast-associated scarring, enhanced transparency, and increased intraepithelial nerve regeneration by approximately 50% compared with control mice [41]. These findings suggest a potential role for heparan sulfate analogs in promoting epithelial healing and corneal reinnervation after injury [41]. Although conceptually attractive, their specific contribution to nerve regeneration, as distinct from general epithelial healing, remains incompletely defined, and current evidence remains preclinical or early translational.

#### Limitations and Future Directions

Despite encouraging findings, the evidence supporting current and future approaches remains heterogeneous. For amniotic membrane, available clinical data are limited by small sample size, short follow-up, product variability, and uncertainty regarding whether observed nerve recovery reflects direct neurotrophic signaling, anti-inflammatory effects, matrix support, or secondary improvement in epithelial health.

For blood products, findings are encouraging, but most studies remain uncontrolled or small, and standardization across blood-derived products is limited. In addition, cost, preparation logistics, and inconsistent reimbursement limit widespread adoption. Another limitation of topical therapy is bioavailability and retention. Conventional eye drops are limited by tear dilution, rapid clearance, short ocular surface retention, and poor corneal penetration.

### Anti-inflammatory Drugs and Immunomodulators

A controlled inflammatory response is necessary for corneal nerve repair, but excessive or sustained inflammation promotes immune cell recruitment and release of cytokines such as IL-6 and TNF-α, thereby impairing regeneration [43, 44]. This makes inflammation a rational therapeutic target in certain cases of corneal nerve dysfunction.

#### Mechanism of Action

Immunomodulatory therapies aim to restore ocular-surface homeostasis primarily by attenuating maladaptive CD4+ T-cell–mediated inflammation. Experimental evidence indicates that CD4+ T cells and complement C3 contribute to context-dependent corneal sensory neuropathy, identifying these pathways as potential therapeutic targets [45].

#### Available Therapies

Among currently available therapies, cyclosporine A is the best-established anti-inflammatory agent with indirect regenerative relevance. It reduces T-cell activation and cytokine release [46] and also increases NGF expression in human corneal epithelial cells through activation of the p38 mitogen-activated protein kinase (MAPK) pathway [47]. In patients with Sjögren disease, treatment with topical cyclosporine A was associated with an increase in corneal subbasal nerve density [48].

Cyclosporine A has also been reported to improve corneal sensitivity after LASIK, although whether this reflects direct neural effects or secondary improvement in surface inflammation remains uncertain [49]. Despite these findings, the evidence base remains limited to relatively small studies, and durable functional recovery has not been consistently demonstrated.

Corticosteroids and nonsteroidal anti-inflammatory drugs may also support corneal repair when used judiciously by suppressing damaging inflammation [33]. However, their benefit is context-dependent, and prolonged use may itself compromise epithelial healing or ocular surface stability. Doxycycline, which has anti-inflammatory and anticollagenolytic properties, has shown benefit in recalcitrant recurrent corneal erosion, particularly when combined with topical corticosteroids [50] but its impact on nerve regeneration is not known.

#### Developing Therapies

Emerging therapies targeting neuroinflammation are of increasing interest. Urcosimod (formerly OK-101), a lipid-conjugated chemerin-derived peptide targeting ChemR23, was evaluated in a randomized, double-masked, placebo-controlled Phase 2a trial for neuropathic corneal pain (NCT06637527). Preliminary reports indicated greater 12-week reductions in pain with 0.05% urcosimod than with placebo (mean visual analog scale [VAS] reduction, 4.2 vs 2.5 points; >50% improvement, 67% vs 33%), alongside favorable quality-of-life outcomes [51, 52]. Exploratory IVCM findings in a very small subgroup showed directional increases in nerve fiber count and length, although the latter did not reach statistical significance (*p* = 0.057) [53]. These findings require confirmation in larger trials. In January 2026, the FDA authorized a single-patient expanded-access Investigational New Drug application for urcosimod 0.05% in a patient with severe neuropathic corneal pain [54]. However, the currently available evidence is based on company-reported clinical data rather than full peer-reviewed publication, and its translational position should therefore be considered preliminary.

#### Limitations

Despite encouraging findings, current evidence for anti-inflammatory and immunomodulatory therapies in corneal nerve injury remains difficult to interpret. Inflammation is heterogeneous and may originate from the ocular surface, lacrimal unit, systemic immune disease, or deeper trigeminal pathways; therefore, surface-directed therapies may not target all locations of inflammation and/or address all mechanisms of nerve dysfunction. In addition, most studies lack biomarker-guided patient selection, making it unclear which inflammatory pathways are being targeted, and which patients are most likely to benefit.

Clinical evidence is also limited by small cohorts, short follow-up, heterogeneous populations, and inconsistent outcome measures. Importantly, post-refractive surgery studies indicate that recovery of nerve morphology may not necessarily translate into parallel gains in corneal sensation because clinical function and nerve structure do not always correlate consistently [55]. Future randomized studies should combine structural nerve imaging with sensory testing, tear or immune biomarkers, epithelial healing outcomes, and patient-reported symptoms to determine whether these therapies produce durable neuroregenerative benefit rather than transient anti-inflammatory improvement.

### Growth Factors

#### Mechanism of Action

Growth factors are central mediators of corneal nerve repair because they regulate cell survival, migration, proliferation, and axonal regrowth. Several molecules have been implicated in corneal wound healing and regeneration, including epidermal growth factor, transforming growth factor-β, platelet-derived growth factor, and CNTF [56–59]. Among these, NGF has the strongest translational and clinical relevance.

#### Available Therapies

Nerve growth factor promotes corneal healing, increases tear production, enhances conjunctival goblet cell density, and improves corneal sensitivity [60]. Recombinant human NGF (cenegermin) remains the only approved topical pharmacologic therapy specifically indicated for NK. In the REPARO trial (NCT01756456), 156 patients with moderate-to-severe NK were randomized to recombinant human NGF (rhNGF)10 μg/mL, rhNGF 20 μg/mL, or vehicle for 8 weeks. At week 8, corneal healing (defined as less than 0.5 mm of lesion staining) was achieved in 74.5%, 74.0%, and 43.1% of patients, respectively, with most treated eyes remaining recurrence-free during follow-up [60]. These results established proof of concept for growth factor-based corneal nerve therapy.

These findings were supported by the US NGF0214 trial, in which 48 patients were randomized to cenegermin 20 μg/mL or vehicle six times daily for 8 weeks [61]. Complete corneal healing was achieved in 65.2% of cenegermin-treated patients compared with 16.7% of vehicle-treated patients. Treatment also reduced lesion size and disease progression, although recovery of corneal sensitivity was less consistent than epithelial healing.

#### Developing Therapies

Ciliary neurotrophic factor supports neuronal survival and differentiation and may contribute to corneal nerve regeneration [60]. In a murine study, topical CNTF accelerated early nerve regeneration compared with untreated controls, although both groups approached baseline nerve density by 8 weeks [62]. These data suggest that CNTF may accelerate recovery kinetics, but clinical translation remains limited, and no human therapeutic program has yet established efficacy.

Pigment epithelium-derived factor (PEDF) is an endogenous neurotrophic and neuroprotective factor, whereas docosahexaenoic acid (DHA) is an omega-3 fatty acid that serves as a precursor for specialized pro-resolving lipid mediators, including neuroprotectin D1 [63]. In diabetic mice, topical PEDF plus DHA increased corneal nerve density and sensitivity, accelerated epithelial wound healing, and reduced inflammatory-cell infiltration [64]. This combination has not yet been evaluated clinically for corneal nerve disease.

#### Limitation and Real-World Challenges

Cenegermin also illustrates the gap between biologic efficacy and clinical feasibility. Although healing rates are compelling, real-world uptake is constrained by cost, access, treatment burden, and incomplete restoration of normal corneal innervation and sensation. Structural improvement does not always translate into full functional recovery, and recurrence may still occur after treatment discontinuation. Whether cenegermin should be used in early-stage (grade 1) disease remains uncertain and warrants further study.

Furthermore, it remains unclear whether one neurotrophic factor, such as NGF, BDNF, or CNTF, is superior for specific patterns of corneal nerve injury, or whether combination approaches are needed. It is also uncertain whether these agents directly restore corneal nerve structure and function, or whether benefits are mediated primarily through improved epithelial healing and a more supportive ocular surface environment. Future studies should distinguish epithelial from nerve-specific endpoints and define optimal timing, dosing, duration, durability of response, and patient selection.

### Stem Cells and Cell-Free Therapies

#### Mechanism of Action

Stem cell–based therapies have emerged as a potential strategy to enhance corneal nerve regeneration through both cellular replacement and paracrine mechanisms. While early hypotheses emphasized direct differentiation into corneal or neural lineages, current evidence indicates that the primary therapeutic effect is mediated through the secretion of bioactive factors that modulate inflammation, fibrosis, and tissue repair [65–67].

#### Developing Therapies

Mesenchymal stem cells (MSCs), limbal stem cells and induced pluripotent stem cells have been investigated for corneal regeneration. In an experimental corneal alkali-injury model, treatment with either autologous activated omental tissue or allogeneic adipose-derived MSCs promoted corneal wound healing [68]. The therapeutic effects of MSCs may also involve paracrine signaling through cytokines, growth factors, and extracellular vesicles that may have an indirect impact on nerves [69, 70]. This potential was highlighted in a rabbit model of corneal nerve injury induced by epithelial debridement, in which adipose-derived mesenchymal stem cells were injected into the corneal stroma [71]. Although injected cells or cellular remnants were detectable centrally for only 3 days and showed early apoptosis, serial in vivo confocal microscopy (IVCM) demonstrated earlier sub-basal nerve recovery, occurring by Day 14 compared with Day 21 in controls. Enhanced nerve regeneration was further confirmed by immunostaining for SMI-312, a pan-axonal marker of phosphorylated neurofilaments, and βIII-tubulin, a pan-neuronal cytoskeletal marker.

Cell-free approaches, particularly extracellular vesicles (EVs) and exosomes, have gained increasing attention as an alternative to cell-based therapy. These vesicles, typically 50–150 nm in diameter, carry proteins, lipids, and ribonucleic acid (RNA) that mediate intercellular communication and tissue repair [72]. Mesenchymal stem cell-derived exosomes have demonstrated anti-inflammatory, anti-angiogenic, and pro-regenerative effects, including enhanced epithelial proliferation, reduced apoptosis, and improved extracellular matrix remodeling [73]. In a preclinical study, mesenchymal stromal cell-derived extracellular vesicles promoted trigeminal neurite outgrowth in vitro and increased β-III-tubulin–positive corneal reinnervation and sensory recovery following epithelial debridement in mice [74]. Extracellular vesicles obtained from three-dimensional stromal-cell cultures demonstrated greater regenerative activity than those obtained from conventional two-dimensional cultures. These findings remain preclinical, and corneal nerve regeneration has not yet been demonstrated in human trials.

#### Limitation and Real-World Challenges

Evidence that transplanted stem cells form functional corneal nerve fibers in vivo remains insufficient. This highlights a broader challenge in regenerative medicine: biologic plausibility and improved wound healing do not necessarily translate into true neural regeneration or restoration of corneal sensation. Much of the potential benefit may be indirect, through reduced inflammation, improved epithelial integrity, decreased fibrosis, and a more supportive trophic environment, rather than direct replacement or repair of corneal nerves.

In addition, several practical and translational barriers remain. Stem cell and exosome-based therapies vary widely in cell source, isolation methods, culture conditions, cargo composition, dosing, delivery route, and manufacturing standards. These differences make it difficult to compare studies or define an optimal therapeutic product. Long-term safety is also incompletely understood, including risks related to immune response, unwanted fibrosis, abnormal differentiation, angiogenesis, or tumorigenicity, depending on the cell type and delivery strategy.

### Neurotization Techniques

#### Mechanism of Action

Corneal neurotization is a surgical approach designed to restore sensory innervation in severe NK. The procedure involves transfer of healthy donor nerves to the anesthetic cornea, either through direct nerve transfer or interpositional nerve grafts such as the sural or great auricular nerve [75, 76]. These grafts provide a source of regenerating axons that can reinnervate the cornea and restore trophic support.

#### Available Therapy

A systematic review and meta-analysis of 14 retrospective or prospective studies, including 191 patients, demonstrated that corneal neurotization significantly improves corneal sensation, logMAR visual acuity, and disease severity, as measured by Mackie staging in patients with congenital and acquired NK (*p* < 0.0001) [77]. Younger patients tend to show greater improvement in corneal sensation, whereas older patients may experience greater gains in visual acuity, suggesting age-dependent differences in regenerative capacity and functional adaptation [77].

#### Limitation and Real-World Challenges

Neurotization also highlights the distinction between structural and functional recovery. While nerve density and sensation often improve, the degree of restoration varies, and full normalization is uncommon. Surgical complexity, donor site morbidity, and the need for specialized expertise limit widespread adoption. There is also substantial variability in surgical approach, including the choice of donor nerve, harvesting technique, routing strategy, and method of nerve coaptation, and the optimal approach remains uncertain. In addition, optimal timing of intervention remains uncertain, although earlier treatment may be associated with improved outcomes [77].

### Physical and Mechanical Stimulation

#### Mechanism of Action

Physical stimulation modalities have been explored as adjunctive strategies to enhance corneal nerve regeneration. Electrical stimulation (ES), in particular, has demonstrated the ability to promote nerve repair through multiple mechanisms, including upregulation of neurotrophic factors such as NGF and BDNF, inhibition of apoptosis, and modulation of the local inflammatory environment [78, 79].

#### Emerging Therapies

Although not yet tested in humans, in a rabbit lamellar-keratectomy model, transcutaneous ES enhanced corneal nerve regeneration and recovery of sensitivity [78]. The responsible molecular mechanisms remain incompletely defined [78]. In addition, stimulation of trigeminal or nasociliary pathways has been shown to enhance tear production and ocular surface lubrication, thereby indirectly supporting corneal recovery [80]. Clinical data, however, remain limited, and standardized treatment protocols have not yet been established.

Iontophoresis represents another modality that uses electrical current to enhance drug delivery across ocular barriers. By facilitating electromigration and electroosmosis, iontophoresis increases tissue penetration of both charged and neutral molecules [81].

Nanotechnology-based ocular drug delivery represents another promising approach to overcome limited corneal penetration by increasing drug residence time and enhancing tissue penetration [82]. Nanocarriers, including nanoemulsions, micelles, liposomes, dendrimers, hydrogels, and nanosuspensions, can improve solubility, prolong ocular surface retention, enhance tissue penetration, and support more sustained delivery of anti-inflammatory, antimicrobial, immunomodulatory, or neuroprotective agents. By improving control of dry eye disease, infection, and inflammation, these systems may support epithelial recovery and create a microenvironment more favorable for corneal nerve regeneration [82].

#### Limitation and Real-World Challenges

Despite promising preclinical data, the clinical interpretation of electrical stimulation and iontophoresis remains limited by uncertainty regarding mechanism, dosing, and relevant endpoints. For electrical stimulation, it is unclear whether observed benefits reflect direct effects on corneal nerves, indirect improvement in epithelial healing, modulation of inflammation, or altered lacrimal functional unit activity. In addition, optimal parameters—including stimulation site, current intensity, waveform, frequency, treatment duration, and timing after injury—remain poorly standardized, making comparison across studies difficult.

Iontophoresis faces additional translational challenges because treatment efficacy depends not only on the electrical current but also on drug charge, molecular size, formulation, tissue penetration, and electrode design. Therefore, positive effects may reflect enhanced drug delivery rather than a direct neuroregenerative effect of electrical stimulation itself. Future studies should define standardized protocols and determine whether these modalities produce sustained corneal nerve regeneration rather than transient improvement in epithelial or inflammatory status.

With respect to nanotechnology-based formulations, it remains unclear whether they will provide meaningful advantages for corneal nerve regeneration over existing approaches. Future studies will need to evaluate their comparative efficacy for nerve-specific outcomes and also consider cost, access, safety, and feasibility of repeated clinical use.

## Future Directions

Future advances in corneal nerve regeneration will likely depend on developing novel molecular therapies and integrating them with existing treatments, advancing targeted delivery platforms, including biomaterial scaffolds, and using precision diagnostics to better match treatment mechanisms to the underlying cause, phenotype, and stage of nerve injury.

First, emerging approaches may complement current therapies. Among these, topical vitamin B12-containing formulations have shown a signal with respect to corneal nerve repair [83]. In a single-center, randomized, double-masked, placebo-controlled pilot trial (NCT03906513), 30 adults with type 1 or type 2 diabetes previously treated with argon laser photocoagulation received topical citicoline–vitamin B12 or placebo three times daily for 18 months [83]. Citicoline is a precursor in neuronal membrane phospholipid synthesis and may support neuronal membrane repair, whereas vitamin B12 may contribute to neuronal metabolism and axonal integrity. At month 18, corneal nerve fiber length density increased by 33% in the active-treatment group versus 15% with placebo (*p* = 0.04). Corneal sensitivity increased from 28 ± 18 to 52 ± 10 mm in the active group (*p* < 0.0001), and the between-group comparison of percentage changes also favored active treatment (*p* = 0.007). These findings provide preliminary evidence of parallel structural and functional improvement, although confirmation in larger multicenter trials is required. Additional preclinical evidence comes from a formulation containing cross-linked sodium hyaluronate, taurine, and vitamins B6 and B12, which enhanced corneal epithelial repair and produced an approximately 5-fold increase in β-III-tubulin immunoreactivity compared with controls, consistent with increased corneal reinnervation [84]. However, corneal sensitivity was not evaluated; therefore, functional recovery remains unconfirmed.

Targeting neuroimmune signaling represents another emerging direction. An experimental study demonstrated that vasoactive intestinal peptide (VIP) regulates corneal epithelial healing, inflammatory responses, and sensory nerve regeneration through Shh-dependent signaling, which is impaired in diabetic corneas [85]. Together with broader evidence linking inflammation and sensory neuropathy in diabetic corneal disease [86], these findings suggest that modulation of VIP or related neuroimmune pathways may simultaneously suppress inflammation and support nerve regeneration. Alpha-melanocyte stimulating hormone (α-MSH) is another compound with potential effects on corneal nerves and has already been shown to reduce ocular surface inflammation in experimental models [87]. Future studies are still needed to determine the optimal delivery method, dose, timing, and phenotypes that are most likely to benefit.

Rho-associated protein kinase (ROCK) inhibition also warrants further investigation. In a preclinical study, the ROCK inhibitor Y-27632 increased sensory neurite growth and branching in vitro and enhanced corneal nerve regeneration following injury in mice, as demonstrated by longitudinal confocal microscopy and immunofluorescence staining [88]. Although these findings support a neuroregenerative effect, the underlying mechanisms, optimal delivery method, and clinical applicability of ROCK inhibition remain uncertain. These examples highlight the range of molecular targets and pathways that may be leveraged, either alone or in combination with existing therapies, to enhance corneal nerve regeneration.

Second, advances in ocular drug delivery—including iontophoretic systems, nanoparticle-based carriers, microneedles, sustained-release formulations, and biomaterial scaffolds—may improve corneal residence, tissue penetration, controlled release, and local bioavailability while limiting off-target exposure.

Gene therapy represents a promising strategy for sustained local delivery of neurotrophic factors and other regenerative mediators to corneal tissue [89, 90]. In diabetic mice, a single intrastromal injection of an adeno-associated virus rh10 (AAV.rh10) vector encoding NGF promoted sustained corneal nerve regeneration, improved corneal sensitivity, and accelerated epithelial repair, with regenerative effects maintained for more than 5 months [91]. Nevertheless, vector immunogenicity, dose control, long-term regulation and reversibility of transgene expression, and regulatory requirements remain substantial barriers to clinical translation.

Third, parallel advances in diagnostic phenotyping and outcome assessment will be essential for clinical translation. Longitudinal in vivo confocal microscopy should be combined with standardized esthesiometry, epithelial disease measures, tear or neuroimmune biomarkers, and patient-reported pain outcomes. This integrated approach may help determine whether morphological nerve regrowth is accompanied by functional recovery and distinguish neurotrophic loss of function from neuropathic pain phenotypes.

A key challenge to be solved is dissociation between structural regeneration and functional recovery. Many therapies improve nerve density or epithelial healing without fully restoring corneal sensitivity or long-term ocular surface stability. Future development of advanced therapies must also address cost and accessibility and establish clear, standardized treatment protocols. 

## Conclusion

Over the next 5–10 years, corneal nerve regeneration will likely move toward multimodal, mechanism-based treatment rather than isolated single-agent therapy. Promising strategies may combine neurotrophic factors, extracellular matrix–supportive biomaterials, cell-derived secretomes or extracellular vesicles, and bioengineered delivery platforms to promote organized reinnervation while limiting fibrosis and chronic inflammation. Treatment sequencing will probably depend on the underlying phenotype: inflammation-dominant disease may require initial control of inflammation to establish a permissive regenerative environment, followed by neurotrophic, biomaterial-based, cell-derived, or stimulation-based therapy. Future studies should determine which combinations are most effective for diabetic corneal neuropathy, NK, postsurgical denervation, and neuropathic corneal pain, recognizing that therapeutic goals may differ between restoring deficient innervation and suppressing maladaptive neuroimmune signaling.

## Declarations

### AI use

Artificial intelligence (AI) tools were used solely to assist with language editing and improving the clarity and readability of the manuscript. All scientific content, concepts, interpretations, conclusions, and intellectual contributions were developed by the authors. The authors reviewed and approved all AI-assisted edits and take full responsibility for the final content of the manuscript.
